# A KNIME Workflow to Assist the Analogue Identification for Read-Across, Applied to Aromatase Activity

**DOI:** 10.3390/molecules28041832

**Published:** 2023-02-15

**Authors:** Ana Yisel Caballero Alfonso, Chayawan Chayawan, Domenico Gadaleta, Alessandra Roncaglioni, Emilio Benfenati

**Affiliations:** 1 Laboratory of Environmental Chemistry and Toxicology, Department of Environmental Health Sciences, Istituto di Ricerche Farmacologiche “Mario Negri”—IRCCS, Via Mario Negri, 2, 20156 Milano, Italy; 2 Jozef Stefan International Postgraduate School, Jamova cesta 39, 1000 Ljubljana, Slovenia

**Keywords:** read-across, workflow, integrated similarity, case study, human aromatase

## Abstract

The reduction and replacement of in vivo tests have become crucial in terms of resources and animal benefits. The read-across approach reduces the number of substances to be tested, exploiting existing experimental data to predict the properties of untested substances. Currently, several tools have been developed to perform read-across, but other approaches, such as computational workflows, can offer a more flexible and less prescriptive approach. In this paper, we are introducing a workflow to support analogue identification for read-across. The implementation of the workflow was performed using a database of azole chemicals with in vitro toxicity data for human aromatase enzymes. The workflow identified analogues based on three similarities: structural similarity (StrS), metabolic similarity (MtS), and mechanistic similarity (McS). Our results showed how multiple similarity metrics can be combined within a read-across assessment. The use of the similarity based on metabolism and toxicological mechanism improved the predictions in particular for sensitivity. Beyond the results predicting a large population of substances, practical examples illustrate the advantages of the proposed approach.

## 1. Introduction

The reduction of in vivo tests to be conducted has become essential in terms of resources and animal benefits. Grouping/category chemicals approaches can reduce the number of chemicals to be tested because the available information can be used to estimate the properties of untested substances. During the last decades, read-across methodologies earned attention as important risk assessment tools for data gap filling [1].

Read-across assesses an endpoint of an untested substance (target chemical) (TC) based on the results for the same endpoint for one or more tested substances (source chemicals) (SCs). In the category approach, the similarity concept becomes fundamental since SC and TC need to be “similar” in the context of structure, properties, and/or activities [2].

According to the Second report under Article 117(3) of the Registration, Evaluation, Authorisation and Restriction of Chemicals Regulation (REACH), by the European Chemicals Agency (in 2014), 75% of all REACH registration dossiers included read-across or category formation methodologies to fill information requirements for higher-tier toxicological studies; the majority of them applied to repeat dose toxicity, which is one of the most challenging assessments in the process of animal test replacement [2].

Many successes have been made in the areas of computational toxicology, achieving good performance for many endpoints, but many issues still should be solved, since the in silico models are surely not perfect, and there are many errors. There are clear advantages since predictions can be made simply starting from the molecular structure; however, the performance is poorer for more complex toxicological endpoints (such as chronic toxicity) and it is not straightforward to appreciate the uncertainty associated with the prediction. The use of read-across can improve the predictivity, within a weight-of-evidence perspective. Aspects such as endpoints, chemical space, and methodologies need to be explored in a deeper way. In this sense, the EU policy and the elimination of animal models to evaluate systemic toxicity for cosmetics ingredients in 2009 led to numerous collaborative initiatives on in silico modeling and read-across [2,3]. Actually, the main driver for the expansion of read-across is legislation [3,4].

Numerous reasons have led to the growth of category formation and read-across applications. The fact that numerous chemicals miss relevant toxicological data, the heavy impact of legislation in non-test methods, and the acceptance of read-across for regulatory purposes have directly influenced the growth of research on the non-testing methods. In addition, the development of tools such as the OECD QSAR Toolbox (https://qsartoolbox.org/, accessed on 5 February 2023) to efficiently access the information, has facilitated the process. Read-across is a clear, simple, transparent, and easily interpretable technique to evaluate the properties of a substance, even for more complex endpoints such as repeat dose toxicity and reproductive effects in humans, based on less data, but with higher quality and robustness [4].

Regarding read-across, the advantages seem to be more than the disadvantages [3,4] as read-across approaches reduce the need to test every endpoint for every chemical. Additionally, the assessment of many chemicals as a category can be more efficient and accurate than the assessment of a single compound. To justify a read-across, structural, physicochemical (PC), and biological similarities are often used. The similarities to be considered for the read-across are not defined because they are endpoint- and target-dependent; however, a list of relevant similarities to be considered to form categories was proposed by Cronin et al. [4].

### 1.1. The Process of Category Formation and Read-Across

The steps to perform a read-across are not exactly defined; some decisional workflows have been suggested in the literature. For example, seven key steps considering a discrete organic chemical were proposed by Patlewicz et al. [5], including (1) Decision context, (2) Data gap analysis, (3) Overarching similarity rationale, (4) Analogue identification, (5) Analogue evaluation, (6) Data gap filling and (7) Uncertainty assessment. Another flow based on six steps has been also proposed as in the case of Escher et al. [1], including (1) Problem formulation, (2) Characterization of the target compound, (3) Identification of source compounds, (4) Evaluation of source compounds, (5) Data Gap filling, and (6) Uncertainty assessment. In our opinion the implementation of this procedure needs expertise, and there is subjectivity associated with the approach. Ideally, the steps to develop a category or analogue approach should be defined on the basis of the Read-Across Assessment Framework (RAAF) [6]. Summarizing the process, it is possible to distinguish some basic steps.

1. **Decision context (also called problem formulation and scenario definition):** This serves to define the purpose and thus the scenario of the read-across prediction: for prioritization, or hazard or risk assessment. The scope and decision context determine the level of uncertainty that can be tolerated [1,5].
2. **Target(s) identification:** the TC must be identified as well as the effect/endpoint. [1,4].
3. **Analogue(s) identification:** The “most appropriate” analogues are chosen based on the TC, the available data for read-across and the endpoint to be predicted [1,4].
4. **Analogue evaluation:** The most suitable candidates should have enough data, especially for the endpoint of interest. The evaluation should consider similarities with a focus on different features. The data quality and availability are decisive at this point [7]. Once this step is performed, a category is defined, and it should be evaluated for consistency [4].
  Each step is somehow dependent and linked to the other. For example, the identification and evaluation of the SCs are interconnected and can become an iterative process, which at the same time leads to an enrichment of the initial hypothesis.
5. **Data gap filling:** Once a category has been obtained, the read-across can be performed. This step is called data gap filling and, in some cases, it may be a simple interpolation of source data. Three main strategies can be followed: (1) a conservative strategy based on a worst-case, that considers the TC toxic as the most toxic SC; (2) in trend analysis, if a clear trend between the activity and the structure/property of the SCs is identified; (3) a nearest neighbor approach, meaning that only the most similar SCs are used to infer the toxicity of the TC. For qualitative read-across, a strategy could be a simple majority vote approach [1,2,4,5].
6. **Uncertainty evaluation:** some guiding documents exist on uncertainty assessment, e.g., the weight of evidence [7,8]. The RAAF also proposes a complementary strategy to address the different sources of uncertainties [6]. Finally, when a read-across is done for regulatory acceptance, all processes related to the prediction should be properly documented.

### 1.2. The Process of Category Formation and Read-Across

Currently, several tools have been developed to perform read-across, however, other combinations of resources can be equally effective offering a more flexible and less prescriptive approach. Computational workflows are an interesting application that allows us to use information extracted from several sources, to be integrated into a single tool. Workflows are easily adaptable and controlled by the users, additionally can be shared and modified, allowing the development of new characteristics. In addition, the features can be included or excluded according to the data requirements. KNIME is a free and open-source platform that can be used for this purpose. This platform includes over 1000 nodes that can be integrated to automatize data analysis and machine learning. For the purpose of read-across KNIME offers an interesting solution for workflow implementation, and the user can incorporate its own chemotypes and databases for the prediction of toxicity [8].

In this paper, we are introducing a workflow to identify analogues for read-across. This workflow identifies analogues based on three similarities: structural similarity (StrS), metabolic similarity (MtS), and mechanistic similarity (McS). This workflow has been applied using a database of azole chemicals with in vitro toxicity data for human aromatase enzymes. Structural and metabolic properties are computed using several cheminformatic tools. Structural alerts for the evaluation of mechanistic similarity are taken from Caballero et al. [9]. The final output is a list of the most suitable analogues to infer the activity of the TC. Additionally, statistical validation of the workflow applying the leaving-one-out method is done. At the end of the paper, some case studies to exemplify the workflow performance and its applicability are discussed.

### 1.3. Adapted Scheme for the Present Work

The present read-across workflow was developed using a dataset of azole compounds and was tested with the validation procedure proposed in Section 3.2. The various resources and components used for the present read-across study are described briefly below.

#### 1.3.1. Constructing Read-Across Workflow

**Figure 1:**
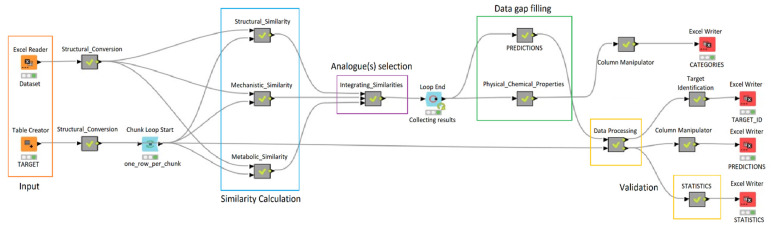
Workflow implemented in KNIME for the automatic integration of structural, metabolic, and mechanistic similarities.

To select analogues for read-across an automated workflow was implemented using the KNIME platform version 4.5.0, adapting and modifying the scheme proposed in Gadaleta et al. [10], see Figure 1. The workflow consisted of the calculation of chemical, metabolic and mechanistic similarities through a search performed in a dataset. The individual list of candidate analogues was retrieved from this dataset considering different kinds of similarities approaches: StrS, a common metabolic behavior, and structural alerts (SAs) to represent the mechanistic similarity. In the next step, the chemicals in the source dataset were ranked considering each kind of similarity. Finally, the output was provided including only the intersection between all the top-ranked compounds. This intersection is then considered as the list of the most suitable analogue (s), and their activity is used to predict the activity of the target chemical [11].

#### 1.3.2. Structural Similarity

**Figure 2:**
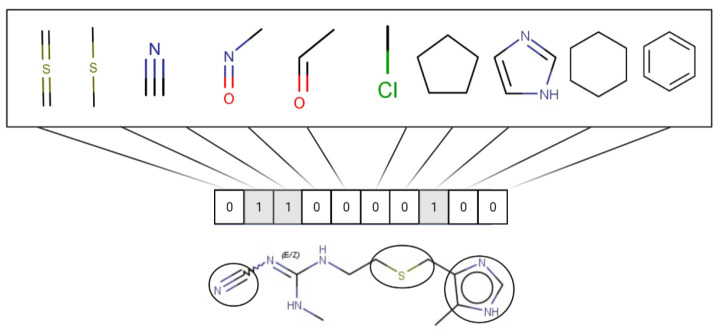
Representation of a hypothetical 10-bit substructure fingerprint with a set of three bits; the substructures they represent are present in the molecule (circled).

StrS was based on PubChem fingerprints [12] that were calculated for both the target and the analogue(s) with the KNIME implementation of the CDK library. The PubChem binary substructures fingerprints can be used for chemical codification and similarity searching, where a substructure is a fragment of a chemical structure, and a fingerprint is an ordered list of binary (1/0) bits. For a molecular fingerprint, each bit represents a Boolean determination for the presence or absence of certain structural properties, for example, an element count, a type of ring system, atom pairing, atom environment (nearest neighbors), etc. Figure 2 represents the hypothetical representation of a 10/bit fingerprint where a set of three bits are indicated, since the substructures they represent are present in the molecule. The native format of the PubChem substructure fingerprint property is binary data with a four-byte integer prefix, where this integer prefix indicates the length of the bit list. Fingerprint computations were based on the CDKit toolkit [13]. PubChem fingerprints are usually applied to calculate structural similarity [14,15]. This kind of codification is useful to disclose analogies for chemical features relevant to biological and toxicological profiles [10].

Furthermore, StrS was measured using the Tanimoto index [15,16]. Tanimoto coefficient was identified in several studies as one of the best similarity metrics for fragment-based similarity searching [15], and is intended as the similarity measure between two points *a* and *b*, with *k* dimensions. Equation (1) illustrates its mathematical definition. The Tanimoto similarity is only applicable to binary variables, and it ranges from 0 to 1, where 1 represents the highest structural similarity. A similarity index of 0.7 was taken as a threshold for the purpose of detecting structurally similar source analogues [17].

(1) $$\frac{\sum_{j=1}^{k}a_{j}\times b_{j}}{\sum_{j=1}^{k}a_{j}^{2}+\sum_{j=1}^{k}b_{j}^{2}-\sum_{j=1}^{k}a_{j}\times b_{j}}$$

#### 1.3.3. Mechanistic Similarity

SAs can be used to evaluate the McS; indeed, it was demonstrated that certain groups or fragments are associated with specific toxic effects [18,19]. Compounds in the source dataset were filtered based on the presence of SAs in common with the target. The 21 SAs implemented within the workflow, were taken from Caballero et al. [9]. In a nutshell, the study identified relevant fragments for human aromatase enzyme activity/toxicity using subsets of azole chemicals and SARPy software (available for download at https://sarpy.sourceforge.net/, accessed on 5 February 2023). Then, the identified fragments were validated and filtered according to their statistical performance to obtain the most relevant and meaningful fragments. In the last step, the study explored the remaining fragments through Structural Activity Relationships to retrieve the final list of fragments, which was considered as the list of 21 SAs for human aromatase activity/toxicity. In the source study from Caballero et al. [9], SAs were obtained from subsets (training sets) of the same dataset used to develop this workflow. Table 1 lists them. The presence of these 21 SAs towards human aromatase binding codified as SMARTS was verified for both the target and the potential analogue(s). For the purpose of the McS, any substances with at least a common SA with the target were considered similar and therefore retrieved for future workflow steps.

#### 1.3.4. Metabolic Similarity

Factors focused on metabolism are proposed through chemical grouping by identifying similar chemicals [2]. In this paper, the metabolism was explored using the WhichCyp package within KNIME [20]. The node predicts which cytochromes P450 isoforms (among 1A2, 2C9, 2C19, 2D6, and 3A4) a given molecule is likely to inhibit. A model output value of “1” indicates the expected binding, while “0” means unlikely to interact, using the output of the model. Binary bit vectors were computed for both the TC and the possible analogue(s) [20]. The generated vectors were utilized to compute the similarities using the Tanimoto index [15,16]; the threshold used to include analogues was 0.7.

#### 1.3.5. Integrating Similarities

For target data gap filling the three independent lists of analogues were integrated using an intersection approach. This means that only analogues that were contained simultaneously in all three similarity lists (StrS, McS, and MtS) were considered for the final prediction. Therefore, the number of analogues used for prediction may vary based on the degree of overlap of the similarity lists. For example, after the threshold application, the StrS list and the MtS list could be formed by the *x* and *y* number of candidates to analogues respectively. While the list of candidates from McS will be formed by any substances with at least a common SA with the target, *z* number of analogues. Then the workflow identifies the intersection between the *x*, *y*, and *z* lists (most suitable analogues) and uses it to predict the target activity/toxicity. The integration approach adapts and modifies the scheme proposed by Gadaleta et al. [10]. The same weight was assigned to all similarities to restrict the number of analogues to the most suitable ones. Therefore, the presence of a chemical in more than one list was interpreted as a higher level of similarity. The prediction of the activity/toxicity was made following a majority vote approach of activities of selected analogues. In the present case, all substances selected according to the similarity criteria, as described above, were used. Conversely, the approach used by Gadaleta et al. had a maximum number of similar substances to be used, according to the similarity metrics [10]. As discussed in the Results section, our approach had a focussed applicability domain, thus the methodology did not process most of the substances to be evaluated. For this reason, we preferred not to impose further restrictions on the number of similar compounds.

## 2. Results and Discussion

### 2.1. Workflow Performance

Computational toxicology is exploiting several pieces of information, present in the datasets and within explicit knowledge which may be codified in SAs, for instance. Within the QSAR models, these pieces of information are typically used to get the prediction, but their role is not always interpretable, and one of the criticisms of many in silico models is that they are opaque. Conversely, read-across is closer to the expert practice, since the use of experimental data, and thus observations, is at the basis of the approach. In the case of read-across, one of the difficulties is to cope with multiple criteria to identify similar substances. In previous studies we introduced the use of multiple similarity metrics, to better explore the source compounds and to identify the relevant ones, limiting the bias of a singular perspective [21]. Here, we further elaborated the approach introduced by Gadaleta et al., skipping the step of the limit to a defining number of similar substances, using different tools to evaluate the structural and metabolic similarity, and applying the new scheme to a new endpoint. For this reason, also the toxicological and mechanistic similarities had to be changed. The new workflow for analogue identification is shown in Figure 1. It was applied to the database for human aromatase binding.

We compared the results using multiple similarity metrics or only StrS. As in Table 2, integrating the three-similarity 92% of the compounds with active properties on the enzyme were correctly classified, and accuracy was 84%. So, based on the leave-one-out (LOO) method, we can expect that at least 88% of the new substances will be correctly classified. Details of the classification performance are provided in Table 2 and the Supplementary Material Files S1 and S2. Worse results were observed using the single StrS. IntS gave higher sensitivity, accuracy, and MCC, indicating overall higher performance. The StrS gave higher specificity, but we observe that from a regulatory point of view, it is preferable to have a low number of FN than FP (see Section 3 for the details on the statistical methods). These observations indicated that the sensitivity was dependent on the three similarities: MtS, McS, and StrS. The overall performance for the truthfulness of the prediction by the system was estimated by the MCC coefficient, which was 0.49 and 0.77 for the structural similarity approach and integrated approach respectively. MCC has been classified as a more informative and truthful score for evaluating the binary classifications than accuracy [22]. MCC is useful in the case of unbalanced datasets, as in the present case.

Overall, a good performance was obtained for most of the chemical categories identified with the IntS. However, the unpredicted rate was much higher (0.79), which indicates the reduced applicability domain of the integrated approach. Indeed, in this case, the requirements are higher, since the same chemical has to be present among the most similar compounds according to all the three criteria for similarity.

The obtained results demonstrate that the use of analogue (s) with diverse kinds of similarity enhances the performance compared to the sole use of StrS. This appears in particular when the SCs used for read-across share more than one SAs with the TC; in this regard, they can be considered mechanistically closer to the TC.

The SCs included in the multiple similarity lists are more likely to match the activity of the TC. Indeed, the combination of all three similarity lists showed the lowest ratio of SCs having a different activity compared with the target, decreasing this ratio more than 3-fold with respect to the sole use of StrS. It is an important highlight, that these are non-unique counts, this means that, if to predict the activity of three different categories, a certain SC was included, this SC will contribute three times to the total number of SCs matching/not matching the target activity. Table 3 presents the number of SCs (non-unique) matching/not matching the target activity, along with the ratio of those not matching the target’s activity; additional data is also provided in the Supplementary Material Files S1 and S2.

### 2.2. The Read-Across Case Studies

To better illustrate the use of the approach, we analyze some case studies, using two specific substances. Table 4, related to the first substance, 1-Hexadecyl-3-methylimidazolium, shows that the IntS approach was better than the StrS alone (see the Supplementary Material Files S1 and S2 too). A total of 11 SCs were identified to predict the activity of the known active compound 1-Hexadecyl-3-methylimidazolium (CAS 61546-01-8) (Table 4). Out of those 11 SCs, 1,3-Didecyl-2-methylimidazolium (CAS 70862-65-6), 1-Methyl-3-octadecylimidazolium (CAS 219947-96-3), and 1-Methyl-3-tetradecylimidazolium (CAS 171058-21-2) were included in all three similarity lists. These three SCs were top-ranked chemicals for StrS, occupying the 4th, 1st, and 2nd positions respectively, however, other eight analogues were also recognized for StrS out of which three were active compounds and five inactive ones (see Table 4).

The metabolic and mechanistic similarities demonstrated the need for extra components for identifying the most suitable analogues. Indeed, all inactive analogues from StrS were excluded from the list of IntS and the MtS played an important role. For instance, Table 5 shows the metabolic pattern for the TC with respect to the five CYP isoforms, i.e., 2D6 inhibition and absence of other interactions with isoforms 1A2, 2C9, 2C19, and 3A4. For example, in Table 5 it can be seen how the 1-Hexyl-3-methylimidazolium, obtained from the StrS list, differed in the metabolic pattern with respect to the TC, while the 1-Methyl-3-tetradecylimidazolium showed a perfect concordance in the metabolic behavior with that of the target.

Considering the McS, the structural alert (SA2_alkyl imidazolium derivatives) was crucial to screen the analogues. For example, SA2 allowed us to discard the 1-Hexyl-3-methylimidazolium ion derivatives, even though it exhibited a high structural similarity with the target (~0.95). This finding agreed with the analysis reported in [9], where a lateral chain of at least 8 carbon atoms was necessary to observe activity on the CYP19A1 enzyme [9].

The examined case study not only constitutes a pragmatic example of how the integration of similarities could represent an advantage for identifying analogues for read-across but also exhibites a high degree of consistency between the obtained category and the literature studies reported [9,23,24,25,26,27,28].

A similar observation was noticed when the TC was the active compound 2-Amino-6-ethoxybenzothiazole (CAS 94-45-1). Table 6 shows that the target was correctly predicted by both RAX approaches: three compounds were selected by the IntS: Methabenzthiazuron (CAS 18691-97-9), Riluzole (CAS 1744-22-5), and Tioxidazole (CAS 61570-90-9). The results of the two approaches were 3 active and 0 inactive compounds for IntS, while there were 6 active and 3 inactive compounds for StrS.

As in the previous case study, the metabolic component included in the IntS allowed us to reject all the inactive chemicals obtained from the StrS list, including the 2-Amino-4-methoxybenzothiazole, a compound that presented a high structural similarity (0.90) with the target (see Table 6 and Table 7). These inactive chemicals removed by the IntS approach showed substitutions at position 4 of the 2-aminobenzothiazole scaffold, which has been associated with inactive chemicals on human aromatase, according to a study reported in the literature [9]; this study captured how the substitution of the 2-aminobenzothiazole scaffold at position 4 leads to inactive chemicals.

Moreover, the IntS demonstrated a higher performance against the use of the StrS alone in many other cases. For example, when the TC was the inactive compound Paclobutrazol (CAS 76738-62-0), the StrS approach produced a category with 13 SCs, all of them with a high level of StrS to the target. They were 10 active and 3 inactive chemicals, leading to a false positive prediction using the majority vote approach. Conversely, the inclusion of metabolic and mechanistic components within the IntS reduced the number of analogues to a unique suitable compound, the inactive chemical Triticonazole (CAS 131983-72-7).

## 3. Materials and Methods

### 3.1. The Dataset on Aromatase

To verify the use of the new workflow for read-across, we used a dataset of 326 azole compounds with experimental values on human aromatase breast cancer cell line (MCF-7aro, cell-based assay). The starting dataset was initially collected from the Tox21 library considering only Tox21_Aromatase_Inhibition (activity test). This contained 20,992 compounds encoded as SMILES, name, and CAS number [29]. The assay was performed using aromatase breast cancer cell line (MCF-7 aro) (cell-based assay) and the concentrations of testosterone (an androgen and estradiol (an estrogen)) were measured before and after exposure to azole compounds tested. The qualitative outcome was recorded as an active agonist, active antagonist, and inactive, where quantitative agonist and antagonist activities were expressed in nanomolar (nM) units represented by AC50 in the original database [29]. Once the data was collected, it was subjected to a rigorous data curation procedure. The first step involved the retrieval of SMILES following the workflow developed by Gadaleta et al., 2018 [30]. The maximum purity was labeled “A” and only compounds with this label were considered. The detection of inorganic compounds, organometallic compounds, mixtures, neutralization of salts, tautomeric forms, and chemotype normalization was performed using the KNIME platform [31]. The compounds with inconclusive assay outcomes were discarded and duplicate structures were classified into two cases as follows: (i) activity range lower or equal to 1:3, and (ii) activity range higher than 1:3. In the first case, the mean of the activity was calculated, and in the second case, the structures were rejected. 3459 compounds were kept from the original dataset which had the purity “A” label. Furthermore, 67 compounds with ambiguous values, 10 compounds with trace element or inorganic compounds, 3 mixtures, 6 duplicates, and 6 ionic liquid compounds were removed. After this, the dataset was subjected to a manual inspection process and 119 compounds were found to have incorrect structures, and therefore removed. At this point, the dataset contained 3248 compounds and was filtered to extract azoles only. The total number of azoles was 351, from them 25 were tetrazoles and were discarded due to their poor representation. The quantitative outcome in nanomolar (nM) units was converted to molar (mole/liter) using the formulae (−logAC50 + 9). The qualitative activity values, active agonist and active antagonist, were recorded as “active”. The distribution of compounds in the final dataset of 326 azoles, considering the numbers of nitrogen in the azole ring and their qualitative activity value was:

- 82 monoazoles compounds of which 61 were inactive and 21 active.
- 198 diazoles of which 148 were inactive and 50 active.
- 46 triazoles which contained 26 inactive and 20 active.

More details regarding the data collection and data curation process are available in Caballero et al. [9,11].

### 3.2. Validation

The presented evaluation process has been validated by predicting chemicals in the source dataset, as in Section 2.1, using the LOO method. Additionally, predictions were performed using only the StrS. A similarity index of 0.7 was taken as a threshold to detect a realistic initial number of structurally similar source analogues [17].

To assess and compare the performance of the approaches, a consistent selection of performance statistics has been chosen and used throughout this work. These are outlined here. Any prediction can be split into four categories, as shown in the confusion matrix as in Table 8 [32].

Based on this confusion matrix, several statistical parameters, such as Accuracy (Acc), Sensitivity (Se), Specificity (Sp), and others, can be calculated. The predictability and reliability can be described by the statistical parameter Acc, which was calculated as shown in Equation (2). The accuracy can take values in a range of 0–1, while the values close to one were desired and interpreted as a better performance during the classification [32,33].

(2) $$Accuracy=\frac{True\;positives+True\;negatives}{Positives+Negatives}$$

True Positives (TP) and True Negatives (TN) represent the number of accurate predictions irrespective of whether the predictions were active/inactive or agonist/antagonist. The sum of positives and negatives represents the total number of predictions made. Other statistical parameters, namely, likelihood ratio, sensitivity, and specificity as shown in Equations (3)–(5) respectively, were also considered to assess the performance. The LR value provides a measure of accurate predictions considering the distribution between classes of compounds, e.g., active and inactive compounds, and the ratio of true and wrong predictions. The ideal value of this parameter is “infinite”, which means that the number of wrong predictions is zero. Hence, the higher the LR value, the greater the contribution towards a single activity class. However, unlike accuracy where the numerical range is well defined, the wide numerical range of LR values is more difficult to interpret.

Parameters such as sensitivity (Equation (4)) and specificity (Equation (5)) were applicable to calculate a measure of the proportion of TP and TN with respect to the total number of positives or negatives respectively [33]. Additionally, the Matthews Correlation Coefficient (MCC) was also computed (Equation (6)) as the rate which produces a high score only if the prediction obtained good results in all the four confusion matrix categories (TP, FN, TN, and FP).

(3) $$LR=\frac{True\;prediction}{Wrong\;prediction}\times \frac{Positives\;}{Negatives}$$

(4) $$Sensitivity=\frac{True\;positives}{True\;positives+False\;negatives}$$

(5) $$Specificity=\frac{True\;negatives}{True\;negatives+False\;positives}$$

(6) $$MCC=\frac{\left(TP\times TN-FP\times FN\right)}{\sqrt{\left(TP+FP\right)\left(TP+FN\right)\left(TN+FP\right)\left(TN+FN\right)}}$$

## 4. Conclusions

This paper describes an automated workflow that identifies analogue(s) for read-across. This approach can support toxicologists during the analogue identification step and provide an automated prediction based on the most suitable analogues.

It offers an easily interpretable framework constructed upon diverse similarity considerations for read-across on human aromatase. A key component of this method involves the joint evaluation of the chemical, metabolic and mechanistic similarities between the target and source compounds that helps define the toxicological outcomes of the enzyme. As in conventional read-across, the StrS constituted the starting point for the approach, however, the addition of new components strengthened the evidence in the analogue(s) selection.

The result of this process is a more reliable prediction, together with a list of the most suitable analogue(s) which can be applied for read-across. This approach provides a comprehensive basis for selecting appropriate analogues in read-across for other endpoints; however toxicity is evidently dependent on many variables and any new endpoint should be individually studied using specific information on the mechanism, for instance through SAs.

Overall, the results on the dataset as shown in Table 2, and examples of case studies clearly indicate that the use of the multiple similarity metrics improves the performance of read-across, and the interpretability of the outcome. Both these aspects are quite important. The information on the toxicological mechanism must be addressed specifically for each property, since the SAs or the other ways to codify the toxicological information is peculiar. If we compare the good results obtained both in the present study with what was reported previously by Gadaleta et al. [16], where different methods were used for the StrS and the MtS, we can assume that the general approach to integrating multiple metrics is quite robust, and different procedures to measure the StrS and MtS can be applied.

The link between metabolism and toxicity has been understood for many compounds, as well as its relevance to evaluate similarities and differences, but the uncertainty related to metabolic predictions continued to be challenging. For similarity assessment, another metabolic consideration could be addressed, e.g., same reactivity, metabolic pathway, or bioavailability, but the introduction of new predictions can also increase the uncertainty.

In general, the methodology exhibits a good predictive performance comparable to a QSAR model but suffered from the use of a small database and a restrictive approach, causing several compounds not predicted. StrS and a second similarity parameter can be applied, increasing the number of compounds evaluated, but reducing the accuracy. Considering that this approach was designed to identify the most suitable analogues for read-across, and not to predict large-scale chemical toxicity, we can affirm that the main strength of this integrated read-across approach is its ability to provide reliable and simply interpretable results, joined to appropriate data to support the final evaluation of the toxicity outcome.

The application of this workflow should be case-by-case performed, for experts’ decision regarding the appropriateness of the identified analogue(s) since the same weight was considered for the integration of the three similarities. The outcome of this workflow could be combined with other sources of evidence, such as for example, adverse outcome pathways which have been demonstrated to be an attractive and confident method to identify toxic effects.

## Figures and Tables

**Table 1:** Structural alerts for human aromatase toxicity.

Structural Alert_ID	SMARTS	Associated Toxicity
SA_1	n1c(N)sc2cccc(c12)	Toxic *
SA_2	c1c[n+](cn1CCCCCCCC)C	Toxic
SA_3	N#C	Toxic
SA_4	c1c(cccc1Cl)Cl	Toxic
SA_5	c1ccc(cc1)c1nc(N)sc1	Toxic
SA_6	n1ccsc1C	Non_Toxic
SA_7	O=c1c2c(ncn2C)n(c(=O)n1)C	Non_Toxic
SA_8	O=c1ccnc([nH]1)	Non_Toxic
SA_9	N=C(N)N	Non_Toxic
SA_10	O=C(O)C	Non_Toxic
SA_11	c1ccnn1	Non_Toxic
SA_12	S(=O)c1ccccc1	Non_Toxic
SA_13	O=C(NC)C	Non_Toxic
SA_14	c1nc[nH]c1C	Non_Toxic
SA_15	n1c(nnc1C)C	Toxic
SA_16	C(Cn1ncnc1)C(C)(C)	Toxic
SA_17	O=S(=O)(N)	Non_Toxic
SA_18	n1csc(c1C)	Non_Toxic
SA_19	c1cnc(n1C)C	Non_Toxic
SA_20	O=C(OC)c1cncn1	Non_Toxic
SA_21	N(C)C	Non_Toxic

* Toxic means that the fragment was observed to induce a change in the activity of the enzyme. The toxicity may be caused by agonism or antagonism of the enzyme activity.

**Table 2:** Classification matrix and classification performance metrics of the workflow using StrS and integrated similarities approaches.

		Structural Similarity		Integrated Similarities	
		Predicted			
		Positive	Negative	Positive	Negative
Experimental	Positive	49	35	34	3
	Negative	23	178	5	27
Sensitivity		0.58		0.92	
Specificity		0.89		0.84	
Accuracy		0.80		0.88	
Error_rate		0.20		0.12	
Unpredicted rate		0.04		0.79	
MCC		0.49		0.77	

**Table 3:** Ratio of source compounds (SCs) having a different activity compared with the target (non-unique count).

	Number of SCs Matching the Target Activity		Number of SCs not Matching the Target Activity		Ratio of SCs not Matching the Target Activity
StrS	2050		944		0.46
	active	inactive	active	inactive	
	500	1550	472	472	
IntS	211		26		0.12
	active	inactive	active	inactive	
	44	167	13	13	

**Table 4:** The application of the workflow to 1-Hexadecyl-3-methylimidazolium evaluating aromatase binding.

Name	Structure *	Rank	Activity	StrS	Metabolic Similarity	Common Mechanistic Structural Alerts
1-Hexadecyl-3-methylimidazolium	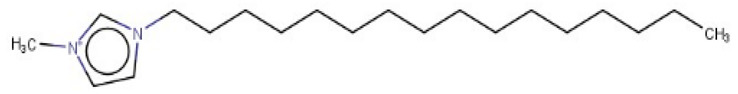	Target	Active	-	-	-
1,3-Didecyl-2-methylimidazolium	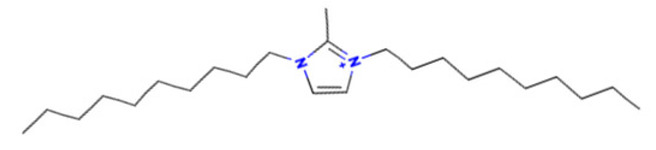	IntS StrS	Active	0.98	1	SA2
1-Methyl-3-octadecylimidazolium hexafluorophosphate	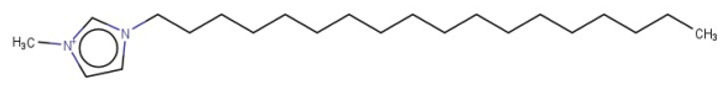	IntS StrS	Active	1	1	SA2
1-Methyl-3-tetradecylimidazolium chloride	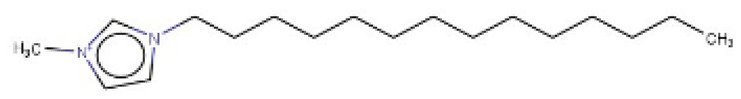	IntS StrS	Active	1	1	SA2
1-Hexyl-3-methylimidazolium chloride	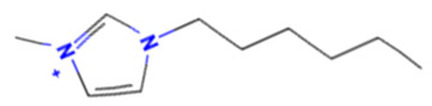	StrS	Inactive	0.95	-	-

7 additional analogue (s) *; (3 actives, 4 inactive); * Analogues identified solely by the StrS approach (full list of analogues is available in the Category table of Supplementary Material File S1).

**Table 5:** The cytochrome isoforms predicted for 1-Hexadecyl-3-methylimidazolium and two structurally similar substances. The isoforms with value 1 are predicted to be inhibited. The fragments which were most significant to predict as binding or non-binding by the CYP isoform are highlighted.

	1A2	2C9	2C19	2D6	3A4
1-Hexadecyl-3-methylimidazolium chloride (target)	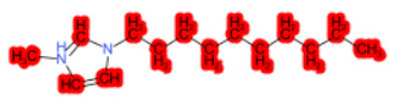	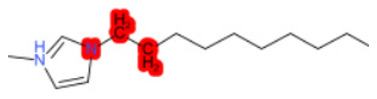	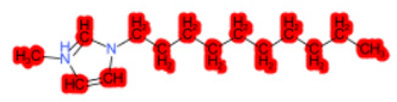	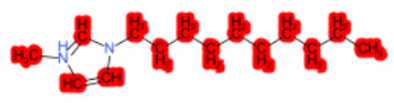	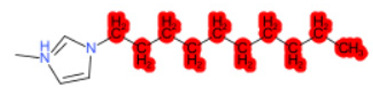
	0	0	0	1	0
1-Hexyl-3-methylimidazolium chloride	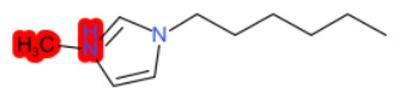	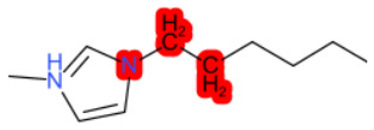	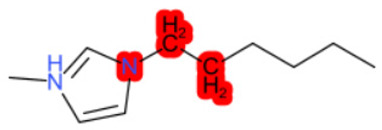	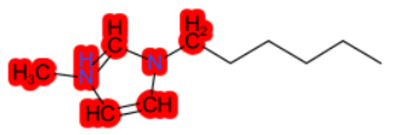	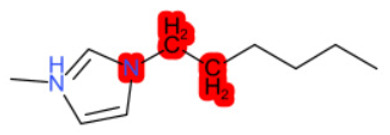
	0	0	0	0	0
1-Methyl-3-tetradecylimidazolium chloride	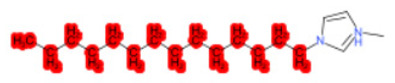	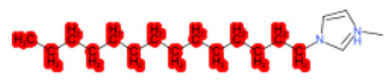	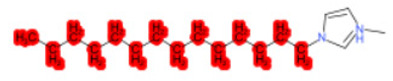	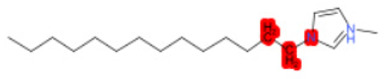	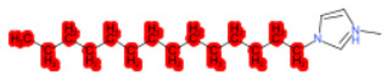
	0	0	0	1	0

**Table 6:** The application of the workflow to 2-Amino-6-ethoxybenzothiazole evaluating aromatase binding.

Name	Structure *	Rank	Activity	StrS	Metabolic Similarity	Common Mechanistic Structural Alerts
2-Amino-6-ethoxybenzothiazole	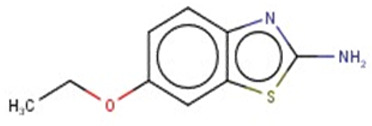	Target	Active	-	-	-
Methabenzthiazuron	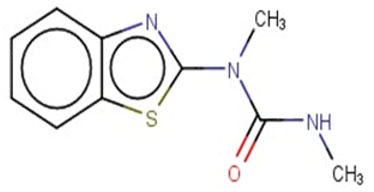	IntS StrS	Active	0.73	1	SA1
Riluzole	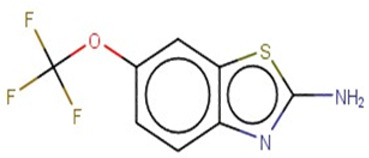	IntS StrS	Active	0.94	1	SA1
Tioxidazole	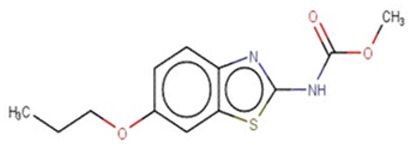	IntS StrS	Active	0.94	1	SA1
2-Amino-4-methoxybenzothiazole	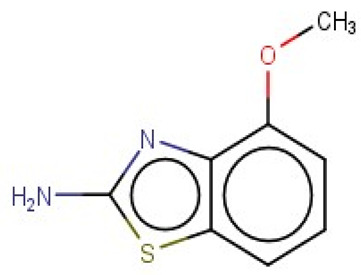	StrS	Inactive	0.90	-	-

5 additional analogues *; (2 actives, 3 inactive); * Analogues identified solely by the StrS approach (full list of analogues is available in the Category table of Supplementary Material File S1).

**Table 7:** The cytochrome isoforms predicted for 2-Amino-6-ethoxybenzothiazole and two structurally similar substances. The isoforms with value 1 are predicted to be inhibited. The fragments which were most significant to predict as binding or non-binding by the CYP isoform are highlighted.

	1A2	2C9	2C19	2D6	3A4
2-Amino-6- ethoxybenzothiazole (target)	1	0	1	0	0
	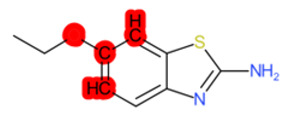	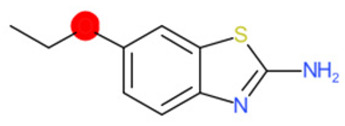	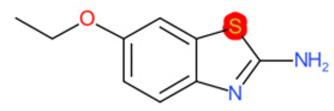	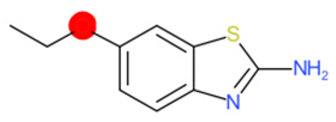	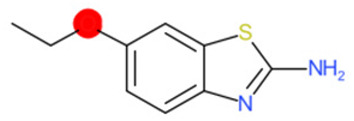
Methabenzthiazuron	1	0	1	0	0
	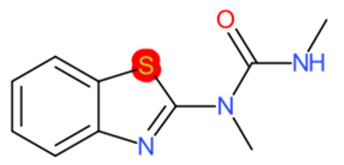	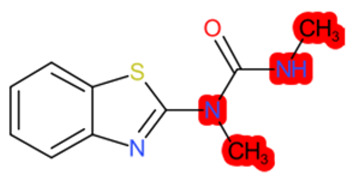	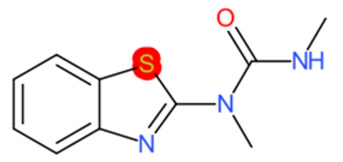	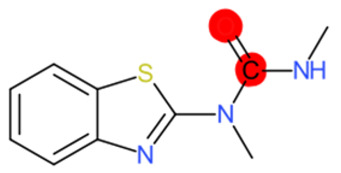	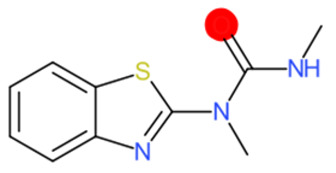
Riluzole	1	0	1	0	0
	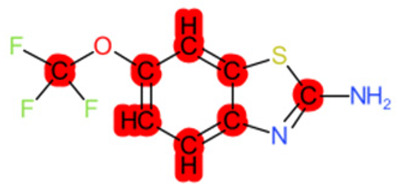	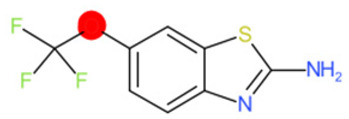	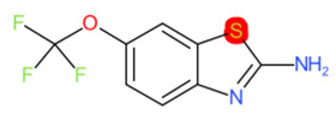	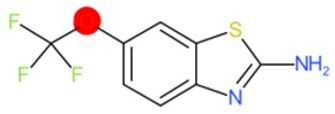	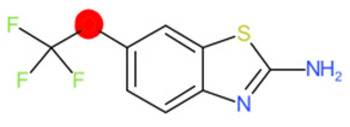
Tioxidazole	1	0	1	0	0
	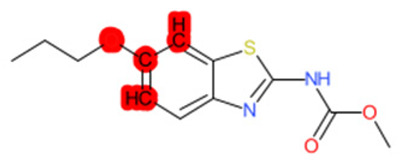	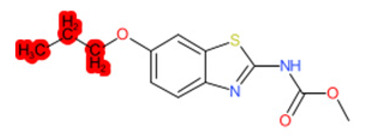	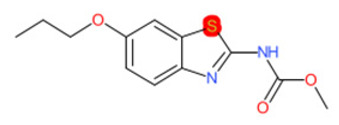	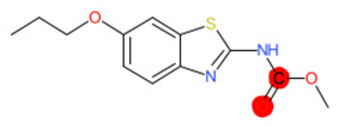	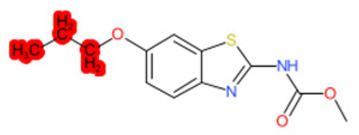
2-Amino-4- methoxybenzothiazole	1	0	0	0	0
	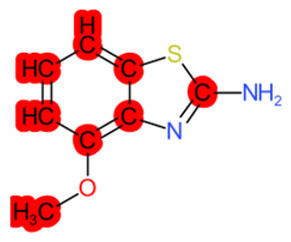	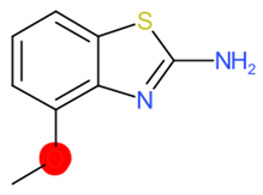	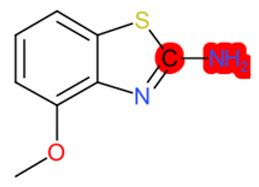	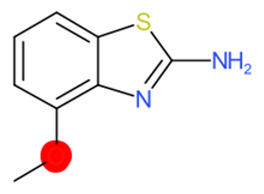	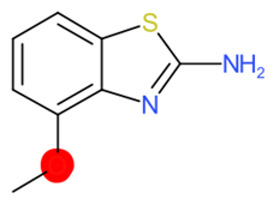

**Table 8:** The definition of true positive, false positive, false negative and true negative.

	Predicted Positive	Predicted Negative
**Experimental Positive**	“True Positive”	“False Negative”
**Experimental Negative**	“False Positive”	“True Negative”

## Data Availability

Not applicable.

## Supplementary Materials

The following supporting information can be downloaded at: https://www.mdpi.com/article/10.3390/molecules28041832/s1, File S1: Structural Similarity; File S2: Integrated Similarity.
