# Supplementary material for: A KNIME Workflow to Assist the Analogue Identification for Read-Across, Applied to Aromatase Activity
Source: Molecules. 2023 Feb 15;28(4):1832. doi: 10.3390/molecules28041832 (PMC9961311; doi:10.3390/molecules28041832)
Supplement: Supplementary file 1 [file molecules-28-01832-s001.zip › User's guide.docx]

A KNIME workflow to assist the analogue identification for read-across, applied to aromatase activity.

Ana Yisel Caballero Alfonso ^1,2,^* , Chayawan Chayawan ^1^ , Domenico Gadaleta ^1^, Alessandra Roncaglioni^1^ and Emilio Benfenati ^1,^*

This is a workflow to support the analogue identification for read-across. The implementation of the workflow was performed using **KNIME 4.5.0**, and a **database of azoles** chemicals with *in vitro* toxicity data for **human aromatase enzyme**. The workflow identify analogues based on three similarities:

**Structural similarity** (StrS): based on PubChem fingerprints

**Metabolic similarity** (MtS): based on mechanistic structural alerts for human aromatase enzyme

**Mechanistic similarity** (McS): based on WhichCyp 1.2 to predict which cytochromes P450 isoforms among 1A2, 2C9, 2C19, 2D6 and 3A4 a given molecule is likely to inhibit.

The read-across **prediction is made based on the intersection of the three similarities** without any future restriction.

INSTALLING THE WORKFLOW

1. Install the KNIME 4.5.0 , downloadable at https://www.knime.com/downloads.
2. Open KNIME.
3. Go to *"File -> Import KNIME Workflow"*.
4. Tick *"Select File:"* and go to *"Browse...”* Select the *.knwf file of the workflow (RAX_workflow).
5. Click to *"Finish”*.
6. The workflow now is in your *"KNIME Explorer"* menu on the left of the screen. Double-click on the workflow to open it.
7. If some of the plugins used for the workflow are missing, a message will appear asking you to install the missing extensions. Click on *"Ok"*. The procedure will guide you in the installation of the missing extensions.
8. Restart KNIME

USING THE WORKFLOW

1. Load the input in the table creator node:

- Double-click on the node table creator, called TARGET.
- Type the Name, CAS number (CAS_NOs), and SMILE, remaining columns may stay empty columns.
- Click *“OK”*.

1. Execute the workflow by clicking on the *“Execute all the executable nodes”* button on the top left button of the window or pressing Shift + F7.
2. Modify some of the parameters (OPTIONAL):

- Double click on a metanode (Ex. Structural Similarity metanode).
- Select the numeric row splitter node named *“similarity higher than 0.7”*, and press F6.
- Set the desired threshold value. By default, is defined as 0.7.
- Click *“OK”.*


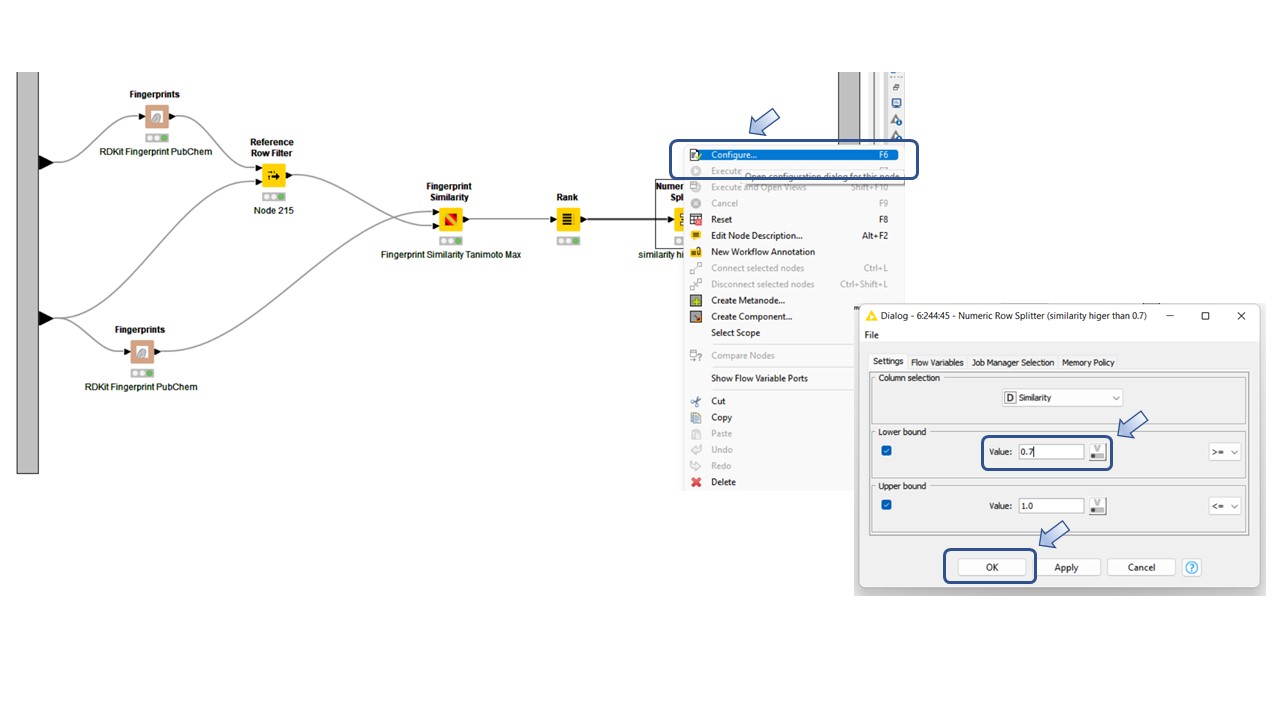


1. Select the path of the output files (by default, the desktop). On each writer node (Predictions, Statistics, PC_Properties, and Secondary Output):

- Secondary click on the node and click on configure or select the node and press F6.
- Select the excel format.
- Specify where you would like to write the file.
- Select the path of the output file. By default, a file is named as the writer, for example: *“Predictions”.*
- Click *“OK”.*


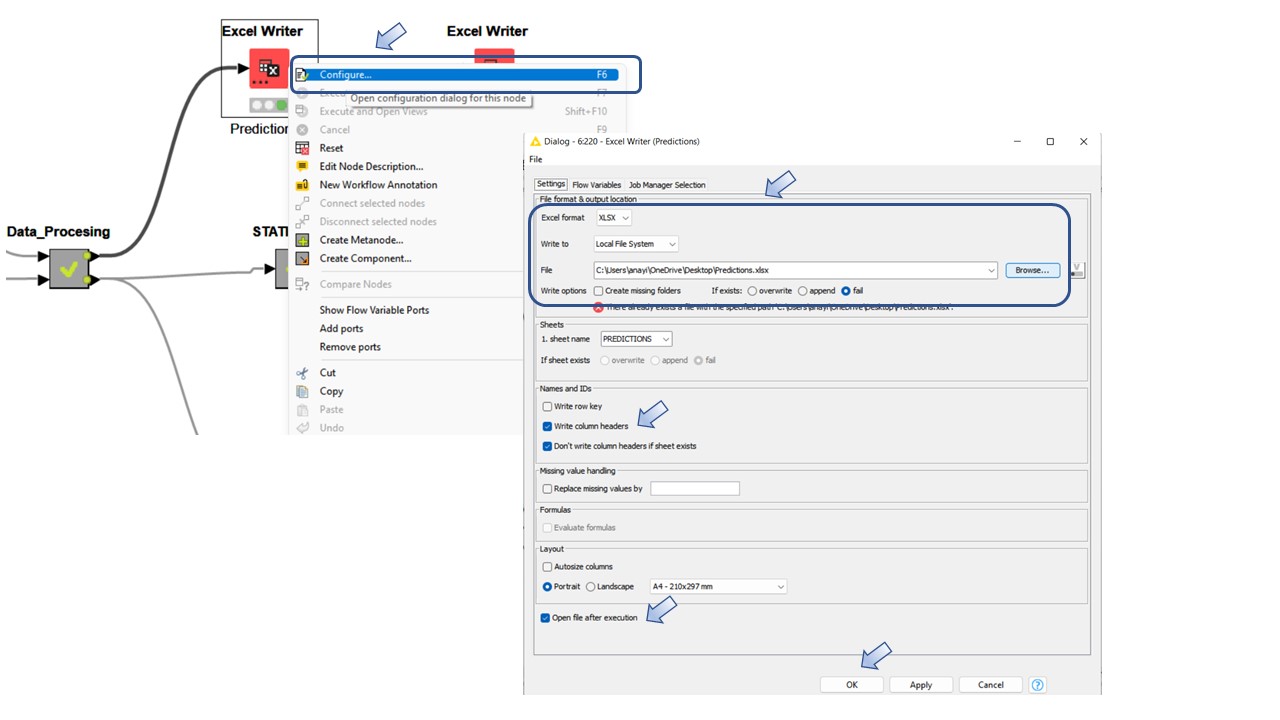


1. Execute the workflow by clicking on the *“Execute all the executable nodes”* button on the top left button of the window or pressing Shift + F7.
2. Press *“Save”* on the top left of the window to save the changes.

HOW TO READ THE OUTPUT

To inspect the RESULTS every file can be checked at the output file (by default the desktop):

1. TARGET_ID: contains the target identification profile, this is:

- The initial information provided by the user.
- The predicted metabolic profile of the target.
- The mechanistic profile of the target is the identified structural alert (when applicable).
- Predicted physical chemical properties of the target (Mannhold LogP, Hydrogen Bond Acceptors, Hydrogen Bond Donors, Topological Polar Surface Area, and Molecular Weight columns).

1. CATEGORIES: contain the list of most suitable analogues after similarity integration, and their profile information, this is:

- The list of analogues within the category, and their information available in the original dataset (Name, CAS number (CAS_NOs), SMILE, and the Assay Outcome (activity) columns).
- The structural similarity index of the analogue compared with the target, and the position of the analogue in the structural similarity rank (Structural_Similarity (Tanimoto) and Structural_Similarity rank columns)
- The structural alert(s) found in common between the analogue and the target (Matched Structural alert column)
- Predicted metabolic profile of the analogue(s), against binding/no binding prediction of the analogue to 1A2, 2C9, 2C19, 2D6, and 3A4, and the similarity measure between the analogue metabolic profile and the target metabolic profile (Binder1A2, Binder2C9, Binder2C19, Binder2D6 and Binder3A4 and Binder_Similarity columns respectively)
- Predicted physical-chemical properties of the analogues (Mannhold LogP, Hydrogen Bond Acceptors, Hydrogen Bond Donors, Topological Polar Surface Area, and Molecular Weight columns).

NOTE: Each Category_ID number corresponds to its respective target chemical ID number. For example, the TARGET_ID number 289, which is the 2-Amino-6-ethoxybenzothiazole, follows into the Category_ID 289, formed by Methabenzthiazuron, Riluzole, and Tioxidazole.

1. STATISTICS: Contain the classification performance metrics of the workflow using the IntS approach (this is applicable only for validation purposes)
2. PREDICTIONS: Contain the predicted activity value for the target (PREDICTION column), and the number of active/inactive chemicals within the category considered for the prediction (No. of active chemicals in the category and No. of inactive chemicals in the category columns respectively). The TYPE of prediction column is only for validation purposes.

WARNING: To adapt this workflow for a new database, we strongly recommend contacting the corresponding authors.

REFERENCE

Further details on the algorithms used to calculate similarities can be found in the reference publication.

CONTACT

Ana Yisel Caballero Alfonso

Istituto di Ricerche Farmacologiche Mario Negri IRCCS

Via Mario Negri 2, 201 56 Milano, Italy

Tel. +39 02 3901 4396 e-mail: anayisel.caballero@gmail.com
